# Alternative translation initiation codons for the plastid maturase MatK: unraveling the pseudogene misconception in the Orchidaceae

**DOI:** 10.1186/s12862-015-0491-1

**Published:** 2015-09-29

**Authors:** Michelle M. Barthet, Keenan Moukarzel, Kayla N. Smith, Jaimin Patel, Khidir W. Hilu

**Affiliations:** Department of Biology, Coastal Carolina University, Conway, SC 29526 USA; School of Biological Sciences, University of Sydney, Sydney, NSW 2006 Australia; Department of Biological Sciences, Virginia Tech, Blacksburg, Virginia 24061 USA

**Keywords:** MatK, Orchidaceae, Monocots, Pseudogene, Chloroplast, Alternative initiation codons

## Abstract

**Background:**

The plastid maturase MatK has been implicated as a possible model for the evolutionary “missing link” between prokaryotic and eukaryotic splicing machinery. This evolutionary implication has sparked investigations concerning the function of this unusual maturase. Intron targets of MatK activity suggest that this is an essential enzyme for plastid function. The *matK* gene, however, is described as a pseudogene in many photosynthetic orchid species due to presence of premature stop codons in translations, and its high rate of nucleotide and amino acid substitution.

**Results:**

Sequence analysis of the *matK* gene from orchids identified an out-of-frame alternative AUG initiation codon upstream from the consensus initiation codon used for translation in other angiosperms. We demonstrate translation from the alternative initiation codon generates a conserved MatK reading frame. We confirm that MatK protein is expressed and functions in sample orchids currently described as having a *matK* pseudogene using immunodetection and reverse-transcription methods. We demonstrate using phylogenetic analysis that this alternative initiation codon emerged *de novo* within the Orchidaceae, with several reversal events at the basal lineage and deep in orchid history.

**Conclusion:**

These findings suggest a novel evolutionary shift for expression of *matK* in the Orchidaceae and support the function of MatK as a group II intron maturase in the plastid genome of land plants including the orchids.

**Electronic supplementary material:**

The online version of this article (doi:10.1186/s12862-015-0491-1) contains supplementary material, which is available to authorized users.

## Background

Maturases are enzymes that splice introns from precursor RNAs. They are most commonly found in prokaryotes and are thought to be the evolutionary splicing ancestor of the eukaryotic splicesome [1, 2]. Maturases have three functional domains: a reverse transcriptase domain, a DNA endonuclease domain and a maturase (RNA binding and splicing) domain, referred to as domain X [1, 3]. Unlike the splicesome, each maturase usually removes only a single target intron [1, 2, 4]. To date, a total of six maturases have been identified in eukaryotic plant cells: four nuclear-encoded, one mitochondrial-encoded [1, 5] and one, *matK*, plastid-encoded [6, 7]. Among these, MatK, the protein product of the *matK* gene, stands out as having the ability to splice up to seven target introns [2], implicating an evolutionary divergence from its prokaryotic relatives to a role more similar to that of the eukaryotic nuclear splicesome.

The *matK* gene is encoded within the group IIA intron of *trnK*^(UUU)^ in most land plants and some green algae [6, 8]. In a few plant species, the surrounding *trnK*^(UUU)^ exons were lost but *matK* was retained as a free-standing gene in the plastid genome. Examples include the fern *Adiantum capillus-veneris* [9], parasitic angiosperms such as the beech-drops *Epifagus virginiana* and some species of the dodder genus *Cuscuta* [10, 11]. There are a few instances, principally in members of *Cuscuta* subgenus *Grammica*, in which the *matK* gene has been lost from the plastid genome [11]. In plants that have lost the *matK* gene*,* the group IIA introns, the principle targets for MatK protein activity, were also lost, suggesting co-evolutionary reduction and supporting the proposed function of MatK as a group IIA intron maturase of the plastid [11].

The proposed function of MatK as a group II intron maturase has gained substantial support through molecular studies that demonstrated expression across a wide range of angiosperms, identified intron targets and generated fine mapping of binding sites [2, 12, 13]. No knock-outs exist for the *matK* gene; however, studies of the ribosomal white barley mutant *albostrians* correlated lack of processing of MatK intron targets with lack of MatK protein [14–18]. Intron targets for MatK activity include introns within transcripts for four tRNAs (*tRNAV*^*(UAC)*^, *tRNAI*^*(GAU)*^, *tRNAA*^*(UGC)*^, and *tRNAK*^*(UUU)*^), two ribosomal proteins (*rpl2* and *rps12*), and a subunit of chloroplast ATPase (*atpF*) [2]. All but one of these intron targets lie within RNAs essential for proper formation and function of the plastid translation complex, suggesting that MatK is a vital component of overall plastid activity. The *atpf* intron target implicates an important role for MatK in photosynthetic activity. It is curious, therefore, that numerous photosynthetic plant species of the second largest angiosperm family, the orchid family (Orchidaceae, monocots) [19] are noted to contain *matK* as a pseudogene [20–23].

To date, there are over 121,000 entries of *matK* gene sequence in GenBank. Of these, 3,094 have *matK* listed as a pseudogene, with approximately 82 % (2,523) of these entries in the Orchidaceae. A pseudogene is a gene that lacks functional protein product [24, 25]. Causes of transition to a pseudogene state include frame shifts leading to premature stop codons and subsequent truncated non-functional protein, decay of coding sequence due to high rate of nonsynonymous substitution, and loss of transcription or processing required for protein translation (reviewed in Harrison et al. [24]). Classification of *matK* as a pseudogene in the Orchidaceae has been based on the presence of frame-shift mutations, specifically non-triplet indels (insertions/deletions), resulting in apparent premature stop codons that form truncated protein [20, 22, 23, 26], as well as a high rate of nonsynonymous substitution [22].

The *matK* gene is considered a rapidly-evolving gene due to a high rate of substitutions at both the nucleotide and amino acid level [21, 27–30]. Substitutions in *matK* are not concentrated in the third codon position but appear to be distributed almost equally among all three codon positions, resulting in a significantly higher nonsynomous substitution rate compared to other plastid genes [29, 30]. This inherent mode and tempo of evolution renders *matK* as an invaluable gene in phylogenetic reconstruction and DNA barcoding [29, 31–35] despite the lingering assumptions of it potentially being a pseudogene.

In light of the importance of MatK in plastid function and its wide use in molecular phylogenetics and barcoding, it is important to examine the pseudogene designation of *matK* in the Orchidaceae. Previous bioinformatic analysis has shown that the amino acid substitutions in MatK across land plants are not random but constrained to maintain the chemical nature and presumed function of MatK [36]. Thus, we focused in this study on the putative frame shift mutations that result in possible truncated protein. We examined 115 *matK* sequences from across the Orchidaceae for plausible alternative initiation codons and assessed their impact on the deduced amino acid sequences. Further, we examined MatK expression and activity in selected orchid species where it was noted as a pseudogene. We also assessed the pattern of initiation codon evolution in the Orchidaceae. We propose that the *matK* open reading frame (ORF) of the pseudogene-labelled GenBank entries in Orchidaceae represent cases where the gene has undergone evolutionary shifts resulting in a potential alternative out-of-frame initiation codon. Translation from this alternative initiation codon alleviates previously described frame shift mutations [20, 22, 23, 26] and restores the ORF.

## Results

### Alignments

**Fig. 1:**
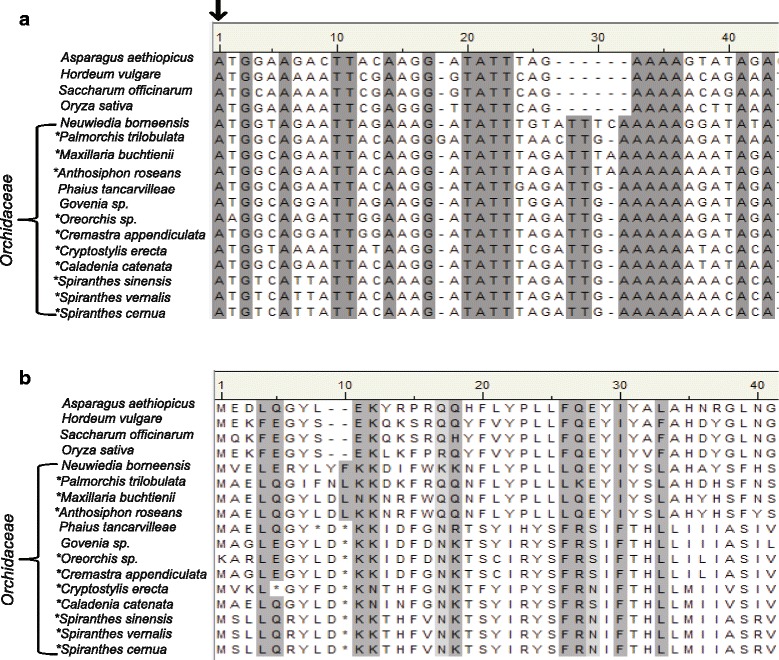
Sample alignments showing the consensus initiation codon (cic) and subsequent translation products for MatK in orchids. (a) Nucleotide alignment of various orchid species and the non-orchid monocot species *Asparagus aethiopicus*, *Hordeum vulgare*, *Saccharum officinarum* and *Oryza sativa*. Asterisks next to species name indicate those noted in GenBank to contain *matK* as a pseudogene. Black arrow indicates cic used by other monocots and angiosperms for MatK expression. (b) Translated MatK amino acid sequence using the cic. Asterisk in translated amino acid sequence indicates stop codons in the MatK reading frame. Gray shadowing indicates highly conserved sequence among all taxa in the alignment, lighter gray indicates less conserved sequence, white background indicates a lack of conserved sequence. Note: gaps in nucleotide and amino acid alignment differ in relative position due to indel in *Palmorchis trilobulata*

**Fig. 2:**
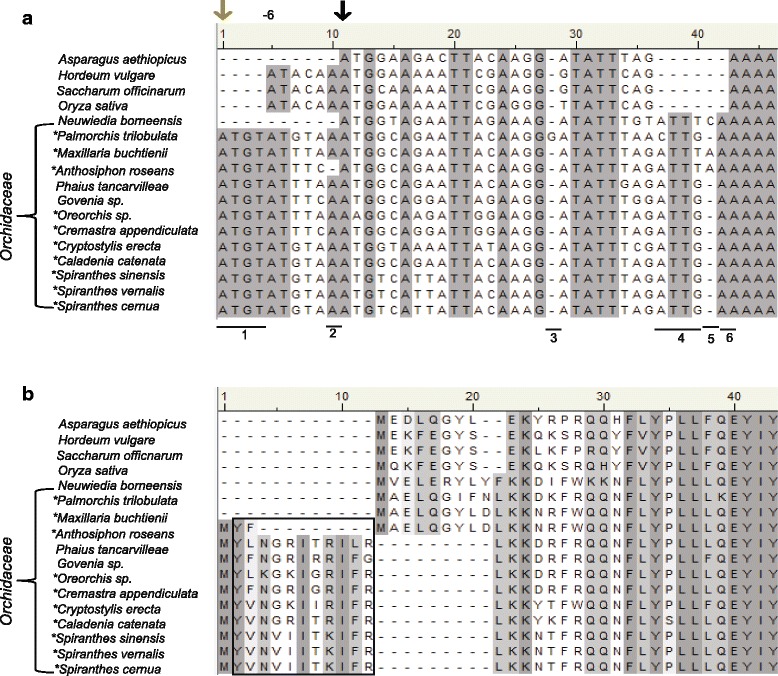
Sample alignments showing the alternative initiation codon (aic) and subsequent MatK translation products. (a) Nucleotide alignment starting at the aic of the same sample orchid species and non-orchid monocot species (*Asparagus aethiopicus*, *Hordeum vulgare*, *Saccharum officinarum* and *Oryza sativa*) as in Figure 1. Asterisks indicate species noted in GenBank to contain *matK* as a pseudogene. Gray arrow shows hypothesized out-of-frame alternative initiation codon. Black arrow indicates consensus initiation codon (cic) used by other monocots and angiosperms for *matK* expression. The ‘-6’ indicates the position of the upstream initiation codon in-frame with the cic. Underlined sections are indels that realign the *matK* reading frame for translation using the consensus vs. the alternative initiation codon*.* Five prime untranslated region of *matK* from *Hordeum vulgare*, *Saccharum officinarum* and *Oryza sativa* shown for contrast in sequence between non-orchid monocots and orchids in this region. (b) Translated MatK amino acid sequence using the aic for species previously containing premature stop codons when translated with the cic (*Phaius tancarvilleae*, *Govenia sp.*, *Oreorchis sp.*, *Cremastra appendiculata*, *Cryptostylis erecta*, *Caladenia catenata*, *Spiranthes sinensis*, *S. vernalis* and *S. cernua*). Asterisks in translated amino acid sequence indicates stop codons in the MatK reading frame. Large gaps in N-terminus of MatK amino acid sequence are indicative of the 11 amino acids different between translations using the aic vs the cic, not true indels. The 11 amino acids that differ due to translation initiation codon are boxed for clarity. Gray shadowing indicates highly conserved sequence among all taxa in the alignment, lighter gray indicates less conserved sequence, white background indicates a lack of conserved sequence

Two data sets were generated, one for the molecular and the other for the informatics aspects of this study. In the former case, GenBank nucleotide sequences were obtained for 13 orchid and four related monocot species representing cases where *matK* was considered as either functional or a pseudogene (Table 1). Translation of *matK* sequence from the initiation codon used for other monocots (referred to hereafter as the consensus initiation codon, cic) produced full length amino acid sequence for MatK protein from the monocot species *Asparagus aethiopicus , Hordeum vulgare, Saccharum officinarum,* and *Oryza sativa* , and three orchid species (Fig. 1). Amino acid sequence from the remaining ten orchid species when translated from the cic displayed premature stop codons suggestive of truncated, non-functional protein (Fig. 1b). Two additional initiation codons were identified upstream of the cic. The first one located −6 bp upstream and in-frame with the cic, was found only in six of the thirteen orchid taxa examined in this data set (Fig. 2). The second upstream initiation codon was found ten bases upstream (−10 position) but out-of-frame from the cic. This second alternative initation codon (aic) was identified in the *matK* gene sequence for all 13 orchid taxa examined and is the result of a four base insertion (ATGT) not found in the non-orchid monocots we examined (Fig. 2a, indel 1). Translation using the alternative initiation codon produced a full-length MatK reading frame for eight of the ten orchid taxa previously reported to contain premature stop codons when translated with the cic (Fig. 2b). A single questionable stop codon was found in MatK of *Anthosiphon roseans* (amino acid position 188) and *Caladenia catenata* (amino acid position 345) when translated using the aic*.* It is to be noted that the cic and the aic in *Anthosiphon roseans* are in the same frame (Fig. 2a).

**Table 1:** Accessions used for alignments in Figs. 1 and 2. Designation of *matK* functionality in GenBank and species taxonomic affiliation are noted

Family	Species	Accession	Designation
Asparagaceae	*Asparagus aethiopicus*	[GenBank: AB646503]	functional
Poaceae	*Hordeum vulgare*	[GenBank: FJ897855]	functional
Poaceae	*Saccharum officinarum*	[GenBank: EU434295]	functional
Poaceae	*Oryza sativa*	[GenBank: AF148650]	functional
Orchidaceae	*Neuwiedia borneensis*	[GenBank: AY557209]	functional
Orchidaceae	*Palmorchis trilobulata*	[GenBank: AJ310052]	pseudogene
Orchidaceae	*Maxillaria buchtienii*	[GenBank: DQ210789]	pseudogene
Orchidaceae	*Anthosiphon roseans*	[GenBank: DQ210903]	pseudogene
Orchidaceae	*Phaius tancarvilleae*	[GenBank: KF673844]	functional
Orchidaceae	*Govenia sp.*	[GenBank: EF525690]	functional
Orchidaceae	*Oreorchis sp.*	[GenBank: EU266420]	pseudogene
Orchidaceae	*Cremastra appendiculata*	[GenBank: EU266421]	pseudogene
Orchidaceae	*Cryptostylis erecta*	[GenBank: AJ310014]	pseudogene
Orchidaceae	*Caladenia catenata*	[GenBank: AJ309997]	pseudogene
Orchidaceae	*Spiranthes sinensis*	[GenBank: HE575508]	pseudogene
Orchidaceae	*Spiranthes vernalis*	[GenBank: AJ310074]	pseudogene
Orchidaceae	*Spiranthes cernua*	[GenBank: AJ543917]	pseudogene

In a broader assessment, we examined *matK* sequences of an additional 104 Orchidaceae species and seven monocot outgroup species; 91 of the orchids required translation from the aic whereas the remaining 13 and all outgroup species could be fully translated with the cic (Additional file 1: Table S1). All orchid species with sequence data extending beyond the conserved initiation codon examined in this study were found to contain both the aic and the cic in the 5’ region of *matK* with the exception of a single taxon, *Neottia nidus-avis* (Additional file 2: Figure S1)*. Neottia nidus-avis* [GenBank: EF079303.1] was a rare exception in the orchids and does not contain the cic or the aic unless RNA editing corrects one of these initiation codons in the transcript. Instead *Neottia nidus-avis* appears to use an initiation codon located −6 bp upstream and in-frame with the cic. The orchid species that utilize the aic display three pertinent indels that allow translation of potential full-length *matK* ORF from this otherwise out-of-frame initiation codon. These indels include the four nucleotide insertion containing the aic (Fig. 2a, indel 1), a four base insertion +37 from the aic (Fig. 2a, indel 4) and a single base insertion +42 from the aic (Fig. 2a, indel 6). Together, these three indels represent insertions of a total of nine base pairs, a triplet, realigning the MatK reading frame to that used in non-orchid monocots and only distorting the reading frame for the region between the first and last indel (Fig. 2a, indel 1 and indel 6, respectively). This distortion results in a total of eleven amino acid changes near the N-terminus of MatK when translated using the aic versus the cic in the Orchidaceae (Fig. 2b).

Other indels of importance include two single base pair insertions for taxa that maintain *matK* translation using the cic. *Palmorchis trilobulata* contains a single base insertion +18 from the cic, which is corrected by single base deletion at +31 (Fig. 2a, indels 3 and 5). Reverse situation of single base deletion/ insertion events are detected at the same positions in *Maxillaria buchtienii* that correct the ORF (Fig. 2a, indels 3 and 5).

### Ribosome Binding Site Analysis

Transcription of the *matK* gene utilizes a promoter that most likely includes at least part, if not all, of the 5’ *trnK* exon that lies close to 1000 bp upstream of the *matK* ORF [12, 13]. The transcript for this gene region would include, therefore, both the alternative and the consensus initiation codons. As such, promoter position would have no influence on which initiation codon was used for translation and, thus, it was not examined in this study.

**Fig. 3:**
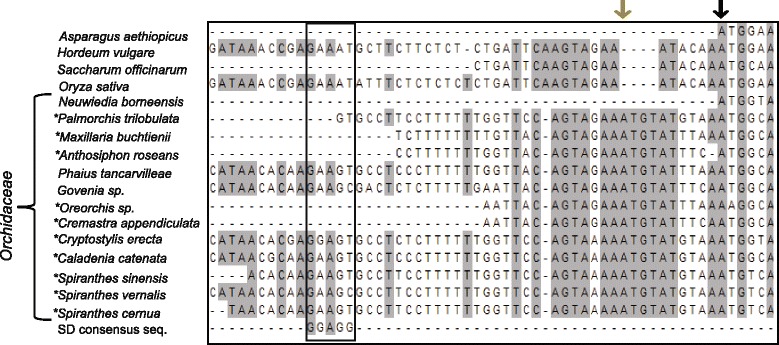
Alignment of 5’UTR *matK* sequence to determine possible Shine-Dalgarno (SD) elements for ribosome binding. Asterisk indicates species noted in GenBank to contain *matK* as a pseudogene. Gray arrow shows hypothesized alternative out-of-frame initiation codon. Black arrow indicates consensus initiation codon used by other monocots and angiosperms for *matK* expression. SD-like region is boxed. SD consensus sequence is shown at bottom of alignment. Gray shadowing indicates highly conserved sequence among all taxa in the alignment, white background indicates a lack of conserved sequence

Aligned sequences of 13 orchids and four non-orchid monocot species were examined for sequence elements suggestive of conserved ribosome binding sites in order to delineate which initiation codon (alternative or consensus) may be preferred for translation of MatK*.* A region with moderate sequence similarity to the Shine-Dalgarno (SD) consensus sequence (GGAGG) used for translation of some chloroplast mRNAs [37] was identified −31 to −28 bases upstream of the alternative AUG in *Phaius tancarvilleae*, *Govenia sp., Cryptostylis erecta, Caladenia catenata, Spiranthes sinensis, S. vernalis,* and *S. cernua* (Fig. 3). No other SD-like sequences were identified in closer proximity to either initiation codon for outgroup monocots or orchids. Other sequence features previously determined to affect translation in chloroplast mRNAs include the −1 triplet [38]. The −1 triplet surrounding the aic varied from AAA to GAA, whereas it varied from C/TAA to TCA for the cic (Fig. 3).

### Protein Expression and Function

**Fig. 4:**
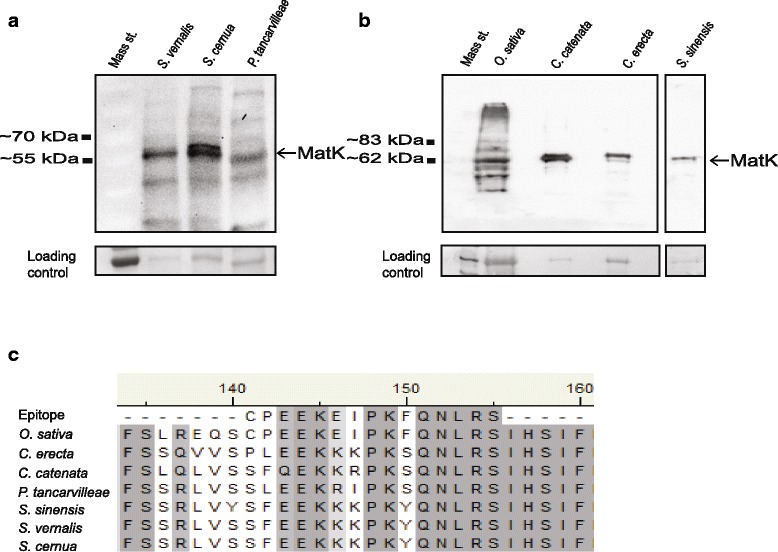
Immunoblot detection of MatK from orchid species. Orchid protein was resolved by SDS-PAGE and transferred to nitrocellulose membrane. MatK protein was detected using anti-MatK antibody as described in Barthet and Hilu [12]. Orchid species analysed represent two different subfamilies of Orchidaceae (Orchidoideae: *Spiranthes vernalis, S. cernua*, *S. sinensis*, *Caladenia catenata* and *Cryptostylis erecta* and Epidendroideae: *Phaius tancarvilleae*) and are representative of orchids that require the alternative initiation codon for full-length MatK translation (Figure 2A and B). All immunoblots were repeated twice to verify results. (a) Immunoblot detection of MatK from 50 μg of total protein from *Spiranthes vernalis, S. cernua and Phaius tancarvilleae*. N = 3 biological replicates. Mass standard = PageRuler Prestained Protein Ladder (Thermo Scientific). Ponceau S stain of RbcS shown as loading control. (b) Immunoblot detection of MatK from 75 μg of total protein from *Caladenia catenata*, *Cryptostylis erecta* and *Spiranthes sinensis*. N = 1 biological replicate due to tissue limitations. *Oryza sativa* (rice) was used as a control for detection. Mass standard = 6–185 kDa Protein Ladder (NEB). Ponceau S stain of RbcS shown as loading control. All immunoblots were repeated twice to verify results. (c) Alignment of MatK peptide region used for antibody generation to orchid species examined in this study

Protein expression of MatK was examined in five orchid species noted to contain *matK* as a pseudogene: *Caladenia catenata, Cryptostylis erecta, Spiranthes vernalis* [39], *S. cernua* [40] and *S. sinensis* [41]. This designation is due to the emergence of a premature stop codon in translations from the cic (Fig.1b). When translation was based on the aic, a full-length MatK reading frame was evident for these same species with the exception of *C. catenata* which is addressed later (Fig. 2a and b). In addition to these five species, we also examined MatK expression in the orchid *Phaius tancarvilleae.* The *matK* gene is noted as a pseudogene in one accession [GenBank: EF079306] but as a functional gene in a different accession [GenBank: KF673844] requiring the aic for full-length translation in the latter accession (Fig. 2a and b). An immune-reactive band ranging from 55 to 65 kDa in mass was observed from Western blots of resolved total protein using an anti-MatK antibody [12] from all six orchid species (Fig. 4a and b). The expected molecular mass of MatK from these orchid taxa is approximately 62 kDa based on amino acid sequence.

Review of Ponceau S staining of RbcS and epitope alignment (Fig. 4a, b and c) demonstrated that discrepancy in signal strength of MatK from Western blots was not attributable to variation in protein loading/transferring to blots or to differences in sequence similarity of the epitope target of anti-MatK. Further there was no evidence of difference in protein degradation on the blots as the reason for the difference in signal strength (Fig. 4a and b). Rather, variation in signal strength may be ascribed to leaf developmental stage at time of collection. MatK has previously been shown to have higher expression during early verses later developmental stages [12, 13]. Tissue samples used for protein analysis were collected from plants obtained through botanical gardens or distributors at various stages of development.

**Fig. 5:**
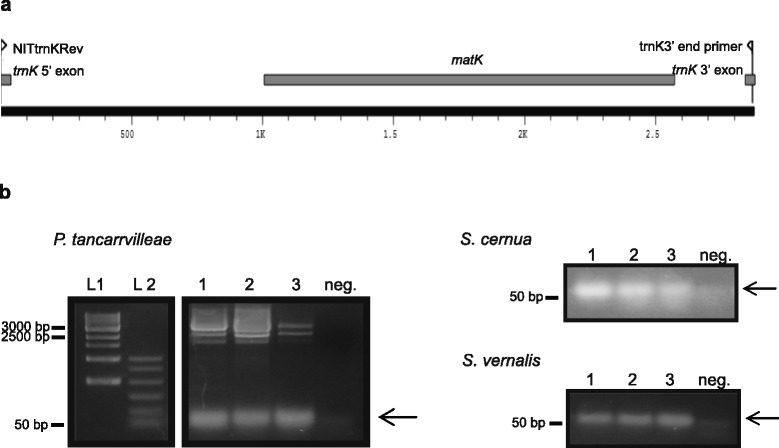
Mature *trnK* ^(UUU)^ product from three orchid species. MatK activity in orchids was determined by examining whether the *trnK* ^(UUU)^ intron was effectively removed from precursor transcript. Total RNA was isolated from three orchid species, *Phaius tancarvilleae*, *Spiranthes cernua* and *S. vernalis* and subsequently converted to cDNA by first strand synthesis using an oligo dT_(20)_ primer. Mature *trnK* ^*(UUU)*^ product resulting from removal of intervening group IIA intron and ligation of surrounding *trnK* exons resulted in a RT-PCR product range from 50 to 61 bp depending on species based on binding position of primers NITtrnkRev and trnK 3’end primer (this study). Precursor RNA still containing intron would result in a band of ~2800 bp based on these same primers. (a) Annotation of primers to the *trnK/ matK* gene region. (b) RT-PCR products using primers specific to the *trnK* ^(UUU)^ exons resolved on 1.7 % agarose gels. Lanes: L1, 1 Kb ladder (New England Biolabs), L2, BenchTop PCR markers (Promega), 1–3 represent products from RT-PCR of three biological replicates for each orchid species examined, neg is the negative control. Sizes of relevant DNA standards based on expected or identified PCR products are shown. Arrows indicate mature *trnK* ^*(UUU)*^ product. Primer dimer due to secondary structure of primers used in PCR is evident as a faint disperse band below the 50 bp standard in negative controls

Pseudogenes are determined by the loss of their ability to make functional protein products [25]. As such, we assessed both the expression of MatK as well as its activity. We examined MatK activity in *P. tancarvilleae, S. cernua*, and *S. vernalis*. These orchid species were chosen based on tissue availability and as representatives of orchid species that require translation from the aic initiation codon (Fig. 2a and b). Reverse-transcriptase PCR (RT-PCR) was used to amplify RNA targets of MatK maturase activity as an indirect indicator of MatK function. Recent analysis of MatK binding to intron substrates using RIPchip assays has identified the intron within *trnK*^(UUU)^ as a primary target for MatK maturase activity [2]. Primers were designed to bind to *trnK* 5’ and 3’ exons (Fig. 5a). Mature *trnK* product (intron removed) based on primer design, would result in a band slightly larger than 50 base pairs (bp) while precursor RNA still containing intron would result in a band of ~2800 bp. Actual *trnK* product sizes in the orchids based on alignments ranged from 50 to 61 bp depending on species. RT-PCR products of a little greater than 50 bp were evident after gel electrophoresis for all three orchid species examined (Fig. 5b). PCR products were sequenced and confirmed as the mature product of *trnK*^(UUU)^. Two additional PCR products of ~2500 and ~ 3000 bp were observed from PCR amplification of *P. tancarvilleae* cDNA (Fig. 5b). These same two products also were observed after PCR amplification of cDNA from some biological replicates of *S. cernua* (data not shown). These larger PCR products are consistent with transcript sizes for the unspliced *trnK/ matK* gene region based on Northern blots [12, 13].

### Phylogenetic Impact of the Alternative Initiation Codon

**Fig. 6:**
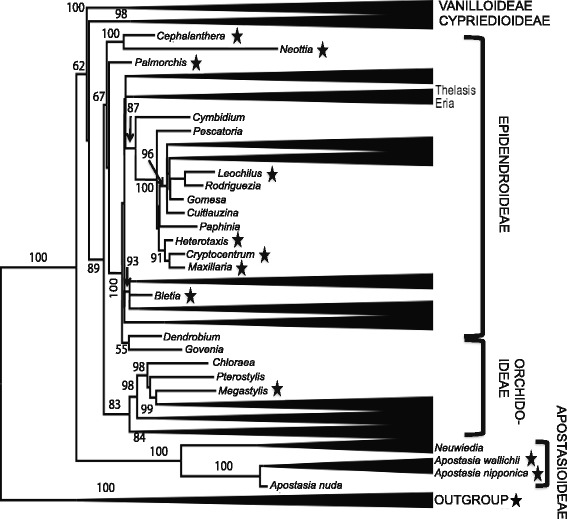
Summarized phylogeny of Orchidaceae determined using *matK* nucleotide sequence alignment based on alternative initiation codon. The tree is rooted with members of the Asparagales monocot families Asteliaceae (Astelia alpina, *Milligania stylosa*), Blandfordiaceae (*Blandfordia grandiflora*), Boryaceae (*Borya septentrionalis*), Hypoxidaceae (*Hypoxis hemerocallidea, Spiloxene serrate,*), and Lanariaceae (*Lanaria lanata*). Asterisks indicate evolutionary lineages that utilize the consensus initiation codon for MatK expression. Lineages lacking asterisks utilize the alternative initiation codon. Relationships were constructed using the maximum likelihood method RAxML in the CIPRES portal (http://www.phylo.org) [70] applying the default settings and conducting 1000 replicates. Bootstrap values are noted on the nodes

Phylogenetic trees derived from two data sets of the *matK* ORF, one aligned using the aic and the second aligned using the cic, are highly congruent with each other. Both phylogenetic trees depict the Apostasioideae being sister to remaining orchids, followed by the Vanilloideae, Cypripedioideae and a clade of Orchidoideae plus Epidendroideae. We opted to use the tree based on the aic for presenting the results and discussing the pattern of *matK* initiation codon evolution. A summary tree is presented in Fig. 6, whereas the detailed tree is presented in Additional file 3: Figure S2. A full *matK* reading frame in all outgroup taxa is obtained based on the cic. Members of the Apostasioideae, the most basal lineage in the Orchidaceae, show full-length MatK translation based on the consensus but not the alternative initiation codon. The exception is *Apostasia nuda* [GenBank: AY557214] that appears to use the aic. In the subsequent diverging lineages, Vanilloideae and Cypripedioideae, all GenBank accessions require the aic for MatK translation. Insertions leading to reversal events to the cic for MatK translation re-emerged later in two orchids subfamilies Epidendroideae (tribes Neottieae, Cymbidieae and Epidendreae), and Orchidoideae (tribe Diurideae) (Figure 6, Additional file 1: Table S1).

## Discussion

Over 3000 GenBank entries across the five Orchidaceae subfamilies have *matK* labelled as a pseudogene with some notable inconsistencies in designation. For instance, two accessions of *Spiranthes sinensis* [GenBank: AB040206 and JF972946] describe *matK* as a functional gene and provide full-length translated amino acid sequence while one accession [GenBank: HE575508] lists *matK* as a pseudogene with no translation. We assessed nucleotide sequence data for over 115 orchid taxa representing all the subfamilies to determine plausible explanations for the perceived pseudogene notations of *matK* in this family. The underlying reason for labelling *matK* as a pseudogene vs. functional gene is the choice of AUG initiation codon used for translation, as protein translation is highly dependent on the frame used. In the accessions where *matK* was labelled as “pseudogene”, we identified an alternative AUG (ATG shown in our DNA alignments) initiation codon ten nucleotides upstream and out-of-frame from the initiation codon used for *matK* translation in other monocots (Fig. 2a). This alternative initiation codon has been overlooked, or possibly avoided since it is out-of-frame with the cic traditionally used in *matK* translation, resulting in inconsistencies of functionality designation. One such example is with the aforementioned *Spiranthes sinensis* [GenBank: HE575508] which lists *matK* as a pseudogene. Although this accession lists *matK* as a pseudogene, the ORF can be translated fully from the alternative initiation codon located at position 37–39 of the submitted sequence (Fig. 2b). Our investigation has demonstrated that the misidentification of the correct initiation codon has led to the predominant labelling of *matK* as a pseudogene in the Orchidaceae and the consequent inaccurate conclusions about *matK* molecular evolution in the family. For example, it has been suggested that *matK* is transitioning towards a pseudogene state in the orchid subfamily Apostasioideae due to translation using the cic [42]. However, MatK can be translated into a full-length amino acid sequence for all members of this subfamily from sequences available in GenBank using either the cic or the aic depending on species (Additional file 1: Table S1).

### Molecular Analysis of MatK Expression in the Orchidaceae

The use of multiple initiation codons, including out-of-frame initiation codons has been previously identified for plant viral mRNAs [43]. In general, translation initiates at the 5’ proximal AUG; however flanking sequence features influence initiation codon choice [44]. These sequence features differ between eukaryotic and prokaryotic mRNA. Eukaryotic mRNAs tend to contain a Kozak sequence to signal the primary AUG for translation initiation while prokaryotic mRNAs contain the Shine-Dalgarno (SD) sequence. Many chloroplast mRNAs include an upstream SD–like sequence [37], reflecting their prokaryotic ancestry. Typically the SD sequence is located approximately −10 bases upstream from the initiation AUG [37]. Examples include the plastid genes *rbcL* and *atpE* which use a SD-like sequence highly similar to that from *E. coli* (GGAGG), and in relatively the same position from the initiation codon in *E. coli* translation (−10 to −6 bases and −18 to −15 bases, respectively) [37]. Other plastid mRNAs, however, have been found to have SD-like sequences anywhere from −2 to −44 bases upstream from the initiation codon [37]. The relative position of the SD sequence in chloroplast mRNAs is an indicator of whether or not the SD sequence is used for ribosome binding. If the SD sequence is in very close proximately to the initiation codon (within a few bases) or much farther upstream, e.g. greater than – 20 bases, the SD sequence may not influence ribosome binding and translation [37]. Other sequence features that tend to affect translation of chloroplast mRNAs include the sequence of the −1 triplet and the sequence of the initiation codon itself [38]. A SD-like region was identified upstream of the *matK* alternative initiation codon of orchids (Fig. 3). The distance from the putative SD region to the alternative initiation codon (−32 to −29) suggests that this region most likely does not influence translation. This same SD-like region in the non-orchid monocot taxa examined resides more than 35 bases upstream from the initiation codon (Fig. 3). It is likely, therefore, that the SD sequence has little influence on *matK* expression in monocots. These results are consistent with previous analysis of regulatory elements for *matK* expression [45]. Using heterologously-expressed MatK in *E. coli,* Zoschke et al. [45] demonstrated that SD-like sequences are not required for *matK* translation. The nucleotide triplet −1 from the initiation codon also has been found to influence translation efficiency for chloroplast mRNAs due to possible extended codon-anticodon interaction [38]. The most essential aspect of the −1 triplet is the nucleotide at the −1 position relative to the initiation codon, with uracil being the preferred nucleotide to enhance anticodon binding [38]. The −1 triplet varied from GAA or AAA −1 of the aic to C/TAA or TCA −1 of the cic in *matK* depending on species (Fig. 3). None of these triplets regardless of initiation codon included a uracil (thymine in our DNA alignments) at the −1 position. Thus, the −1 triplet offers little information for discerning *matK* initiation codon choice*.* We propose, therefore, that other unknown sequence features must influence initiation codon choice for *matK*.

Western blots were used to determine whether MatK is expressed in selected orchid species currently noted to contain *matK* as a pseudogene (Table 1; [39–41]). The immune-detection of protein bands corresponding to full-length MatK protein support that MatK is expressed in these orchid species (Fig. 4a and b). As pseudogenes are also characterized by a lack of function [24], we examined the splicing of the *trnK* intron as an indirect indicator of MatK activity in three orchid species, two of which, *Spiranthes cernua* and *S. vernalis*, are suggested to contain *matK* as a pseudogene [40, 41]. Analysis of the *trnK* region by RT-PCR supports that the *trnK* intron is efficiently removed in both orchid species (Fig. 5b). As no inhibitors for MatK acitivity currently exist, direct *in vivo* assessment of MatK splicing is not currently possible. Studies of MatK-substrate binding by RIP-CHiP analysis [2] and the white barley mutant *albostrains* [16, 17] support that MatK is the most likely factor for splicing of the *trnK*^(UUU)^ intron in this study. Thus, some mechanism must be in place in these members of the Orchidaceae to allow expression of full-length functional MatK protein. Two possibilities exist to explain the expression of MatK from the orchids with *matK* pseudogene designation: 1) another copy of the *matK* gene exists in the orchid genome which lacks premature stop codons or 2) expression occurs using the alternative out-of-frame initiation codon identified in this study (Fig. 2a). Twelve plastid genomes from members of the Orchidaceae have been completely sequenced: *Dendrobium officinale* [GenBank: NC_024019], *Cymbidium aloifolium*, *Cymbidium manni*, *Cymbidium tracyanum, Cymbidium tortisepalum*, *Cymbidium sinense* [46], *Erycina pusilla* [47], *Neottia nidus-avis* [26], *Oncidium* hybrid cultivar [48], *Phalaenopsis aphrodite* [49], *Phalaenopsis equestris* [50], and *Rhizanthella gardneri* [51]. None of these sequenced genomes account for a second copy of *matK* in the plastid genome. Consequently, our data suggest that MatK expression occurs using the alternative out of frame initiation codon identified in this study.

There has been one report of a second copy of *matK* in the plastid genome in members of *Corallorhiza* (Orchidaceae) and closely related genera such as *Oreorchis* and *Aplectrum* [52]. In these genera, one copy of *matK* appears intact, while the other copy, the pseudogene copy, contains a deletion of 380 bp at the 5’ end along with several non-triplet indels leading to premature stop codons and truncated amino acid sequence [52]. Sequences of *Cremastra appendiculata* and *Oreorchis sp.*, both of which are genera closely related to *Corallorhiza*, were analysed in this study and found to utilize the alternative initiation codon for translation of full-length MatK amino acid sequence (Fig. 2a and b). The sequences of *C. appendiculata* and *Oreorchis sp.* used in our assessment [GenBank: EU266421 and EU266420 respectively] lack large deletions in the 5’ end of the *matK* gene and, therefore, are not the pseudogene copy of *matK* in the plastid genome described by Freudenstein et al. [52] but a second copy that contains the aic for translation. Thus, even for genera with a potential second copy of *matK*, the aic appears to be the main initiation codon for translation of full-length MatK protein.

Western blot detection of a 62 kDa anti-MatK immune reactive band from *Caladenia catenata* (Fig. 4b) presents an unusual case in which molecular and sequence data are in disagreement. The longest reading frame deduced from translation of the sole accession for this orchid species [GenBank: AJ309997] is only 344 amino acids long, generated using the upstream aic (Fig. 2a and b). This translation is approximately 160 amino acids shorter than normal *matK* ORF. Translation from the aic of *Petalochilus catenatus* (a synonym for the same species) [GenBank: GQ866570] results in a full-length MatK reading frame. Sequence identity between *P. catenatus* and *C. catenata* is ~100 %, differing in a nucleotide at position +932 which is missing in *C. catenata* relative to *P. catenetus*. Correcting this discrepancy in *C. catenata* eliminates all the premature stop codons, resulting in a full ORF recovery when translated with the aic. The *matK* gene is described as a pseudogene for *C. catenata* [GenBank: AJ309997] but as a functional gene for *P. catenatus* [GenBank: GQ866570]. Therefore, this case points to a possible sequence error and provides a convincing argument against the pseudogene labelling of *matK* in *C. catenata*.

A case similar to *Caladenia catenata* is notable in the *matK* sequence of *Anthosiphon roseans.* The single accession available for this species [GenBank: DQ210903.1] is labelled as pseudogene and displays both the aic and cic in frame with each other, separated by six nucleotides. Translation from either initiation codon revealed one premature stop codon (TAG) at +559 from the upstream aic. When the sequence is aligned with *Maxillaria longipes* [GenBank: DQ210999] of the same tribe, a mutation in this codon position from TTG to TAG is apparent. Sequence identity between these two species is 99 %, remarkably high for species belonging to two different genera. This degree of identity argues strongly against *matK* being a pseudogene in *A. roseans* as mutation rates in non-functional genes are exceedingly high due to relaxed selection pressure on them [53]. Thus, the premature stop codon here is either a sequencing error or possibly is subjected to RNA editing. RNA editing has been previously observed in the *matK* transcript from various plant species including the orchids with up to three reported editing sites [16, 54–57].

*Phaius tancarvilleae* presents an unusual case of sequencing differences in GenBank. There are a total of nine accessions for *matK* in *P. tancarvilleae* (Additional file 4: Figure S3). One accession [GenBank: EU490700] is partial, missing part of the 5’ upstream region thus preventing determination of the initiation codon. Two accessions [GenBank: AB040205, EF079306] suggest full-length translation of *matK* using the cic, while all remaining accessions require the aic for full-length MatK expression (Additional file 4: Figure S3). The sequences of the three biological replicates of *P. tancarvilleae* used in this study [GenBank: KP204599, KP204600 and KP2046010] display100% identity with that of [GenBank: KF852707], and require the aic for full-length *matK* translation. Thus, the inconsistency in initiation codon usage for *P. tancarvilleae* implies an apparent variation at the species level, but also could signify a case of sequencing error.

### Pattern of Evolution of the Alternative Initiation Codon

In our extended data set of over 100 orchid species, 13 species were determined to require translation from the cic. To assess the pattern of emergence of the aic, we mapped the aic and cic on a phylogenetic tree derived from our data sets (Fig. 6). This tree is in agreement with the general consensus on the pattern of subfamily divergences in the Orchidaceae, depicting the subfamily Apostasioideae at the base, followed by the subfamilies Vanilloideae, and Cypripedioideae in a grade sister to an Orchidoideae plus Epidendroideae clade [42, 58, 59]). Except for *Apostasia nuda*, the Apostasioideae *matK* sequences display the cic as the appropriate one for translation. The use of the *matK* consensus initiation codon for translation in the first diverging subfamily Apostasioideae and in the closely related sister families, and the prevalence of the aic for MatK translation in the subsequently diverging subfamilies (Fig. 6) provides unequivocal evidence for the evolution of the alternative initiation codon within the orchids. The Apostasioideae includes two genera, *Neuwiedia* and *Apostasia* and a total of 16 species, 10 of them have *matK* sequence representation in GenBank. The Apostasioideae is an intriguing orchid group. Members of this subfamily differ morphologically from most remaining orchids in a number of features, including the lack of the column (gynostemium), a product of fusion of the stamens and the style, which is a prominent feature of the orchids, and the presence of powdery instead of sticky pollen [42, 60]. These traits render the subfamily Apostasioideae as an evolutionary link between the core Orchidaceae and the rest of the order Asparagales where the orchid family resides. Therefore, it fits that the *matK* in the Apostasioideae presents a transitional evolutionary stage from the consensus to the new alternative initiation codon for MatK translation. It is to be noted that *A. nuda* occupies a basal position in the *Apostasia* clade [42]; this study, pointing to an early reversal event from the alternative to the consensus initiation codon in the family.

Multiple reversal events to the cic also appear in the terminal lineages of the Orchidaceae, namely the subfamilies Epidendroideae and Orchidoideae (Fig. 6). It is rather striking that a reversal was first recovered in members of the tribe Neottieae, which emerged at the very base of the subfamily Epidendroideae in our phylogenetic analyses, a position corroborated by the work of Burns-Balogh and Funk [61], Xiang et al. [62] and Freudenstein et al. [63]. All three genera of this tribe (*Cephalanthera*, *Neottia* and *Palmorchis*) require the cic for full ORF translation (Fig. 6, Additional file 1: Table S1). Subsequent lineages in the Epidendroideae reverted back to the aic for MatK translation, but other reversal events to the cic were detected deep in this subfamily in members of the tribe Maxillarieae and in *Bletia* of the tribe Epidendreae (Fig. 6, Additional file 1: Table S1). Therefore, the loss and gain of the consensus initiation codon for MatK translation in the Orchidaceae does not seem to be at random but is confined to specific lineages, e.g. subfamily Epidendroideae and one species in the subfamily Orchidoideae.

The underlying evolutionary drive for the emergence of the aic and the reversals to the cic in the Orchidaceae is not clear. It may reflect possible evolutionary structural/functional constraint on *matK*. More likely, the presence of an additional potential initiation codon is a buffering mechanism required to offset otherwise potential deleterious frequent losses/gains of nucleotides in *matK*. This latter hypothesis is based on the unusually high substitution rate in *matK* [21, 27–30] and the high variability of the N-terminus region of MatK protein in both length and amino acid composition [10, 36, 64]. Further support for this hypothesis is found in the minimal impact of the use of the aic verses the cic in MatK translations. Only 11 amino acids are altered in the N-terminus with aic translations compared to cic translations due to subsequent restoration mutations (four base-pair insertions at +1 and +37 positions and the one base-pair insertion at +42 from the aic (Fig. 2a, indels 1, 4 and 6, and 2b)). The remarkably balanced substitution/indel molecular evolutionary pattern observed in the 5’ region of *matK*, which maintains expression of intact MatK protein, supports the significance of this gene/protein in plastid /plant function. A similar mechanism is evident at the extreme 3’ end of the gene where non-triplet indels have been observed. In these cases, alternate stop codons immediately downstream from the consensus stop codon are present and could be used to terminate translation and maintain ORF integrity [65], again emphasizing the importance of this gene and its protein product for plant function.

## Conclusions

This study has uncovered a unique evolutionary event affecting the expression of *matK*, which is an essential splicing factor in the plastid and a well-utilized gene in plant molecular systematics. Designation of *matK* as a pseudogene has been primarily determined by nucleotide sequence translation starting at an initiation codon identified through alignment to other angiosperms. The *matK* gene in most members of the Orchidaceae has had an evolutionary divergence due to the insertion of four bases upstream from the ‘normal’ (consensus) initiation codon, identified in this study as the alternative initiation codon. Although this initiation codon is out-of-frame with the downstream initiation codon, translation from this upstream initiation codon results in a full-length MatK reading frame with high amino acid sequence identity to the amino acid sequence for MatK from all other angiosperms. In support of these points, we have demonstrated using Western blot and RT-PCR experiments that full-length MatK protein is expressed and functions in sample orchid species currently designated to contain *matK* as a pseudogene. We believe in light of current evidence presented, designation of *matK* as a pseudogene in the orchids needs to be reassessed to address the functional and phylogenetic consequences of this designation.

## Methods

### *matK* Data Sets and Phylogenetic Analyses

We examined 777 *matK* sequence entries of angiosperm from GenBank with a focus on monocots where the Orchidaceae resides. Among these, 104 Orchidaceae species have sufficient 5’ sequence to determine initiation codon suitability. Species chosen for alignment represent all the five subfamilies currently recognized for the Orchidaceae [58,60] with a broader sampling of species from the largest subfamily Epidendroideae. For tree rooting, seven species from five sister families to the Orchidaceae were used: *Borya septentrionalis* (Boryaceae), *Blandfordia grandiflora* (Blandfordiaceae), *Milligania stylosa* and *Astelia alpina* (Asteliaceae), *Lanaria lanata* (Lanariaceae), and *Spiloxene serrate* and *Hypoxis hemerocallidea* (Hypoxidaceae).

Nucleotide sequences for the *matK* ORF were aligned with QuickAlign [66] and translated into amino acids in MacClade [67]. The insertion of gaps in the sequence alignments took into consideration their cost in the homology assessment following Kelchner [68]. The presence of the aic enforced the insertion of gaps that are not in triplets in the region immediately downstream of the initiation codon. Two alignments were generated differing in whether the consensus or alternative initiation codon was used for translation. The data sets were analyzed phylogenetically using the Maximum Likelihood (RAxML version 8; [69] method in the CIPRES portal (http://www.phylo.org) [70] applying the default settings and conducting 1000 replicates. Bootstrap support was calculated for the 50 % majority trees. FigTree v1.3.1 (http://tree.bio.ed.ac.uk/software/figtree/ ) [71] was used to prepare trees for publication.

Alignment of nucleotide and translated amino acid sequence for sample taxa in Figs. 1 and 2 was accomplished using Accelyrs Ds Visualizer software [72]. Accessions used for alignments in Figures 1 and 2 can be found in Table 1. Analysis of ribosome binding sites required the use of longer regions of the *trnK*^(UUU) Lys^ intron and therefore, a different accession was used for *P. tancarvilleae* [GenBank: KP204599].

Accessions of *Phaius tancarvilleae* deposited in GenBank had significant discrepancies in nucleotide alignment with some accessions suggesting translation using the aic [GenBank: KF852707, KF673844, KF673843] and others the cic [GenBank: AB04205, EF079306] (Additional file 4: Figure S3). To delineate the correct sequence for this orchid species, genomic DNA from three biological replicates of *P. tancarvilleae* was extracted using the CTAB method [73]. A region from the *trnK* 5’ exon to approximately 200 bp in the *matK* CDS was amplified using primers BaseEUD200R and trnK3941 [74]. In order to obtain a longer sequence of the *matK* region, the entire CDS was amplified using primers phaiusmatKup5’ (5’-CATAACACAAGAAGTGCCT-3’) and trnK3’endprimer (5’-GGGACTCGAACCCGGAA-3’) (this study). Amplified products were cleaned using the QIAquick PCR Purification Kit (Qiagen) and sequenced. A non-flowering specimen of *P. tancarvilleae* was entered into the Coastal Carolina Herbarium under voucher number: CCUMMB0002.

### Source of Material for Molecular Assays.

Orchid tissue used in this study was obtained through private vendors, botanic gardens or field collection (Additional file 5: Table S2). Confirmation of *Spiranthes* species collected from field was accomplished through sequencing of *matK* cDNA amplified after reverse transcription using the primers (SSFseq: 5’-TTCCATTCTCGTCGCGAT-3’ and SSRseq: 5’-ACGAAGAAACCGAAATAG-3’) (this study). These primers were designed to amplify a region of 369 bp containing at least three mutations specific to individual species of *Spiranthes*. Alignment of sequence from all three biological replicates using Accelrys^©^ DS Visualizer [72] as well as BLAST search in GenBank confirmed field collection of *S. vernalis*. Prior to sequencing, residual components of the original PCR reaction were removed using QIAquick PCR Purification Kit (Qiagen). A flowering specimen of *S. vernalis* was entered into the Coastal Carolina Herbarium under voucher number: CCUMMB0001.

### Protein Extraction/ Western Blotting

Approximately 200 mg of frozen tissue was ground under liquid N_2_ and total protein extracted by addition of Laemmli SDS buffer (62.5 mM Tris, pH 6.8, 2 % SDS, 10 % glycerol and 5 % β-mercaptoethanol) with the addition of 1 mM of phenylmethanesulfonyl fluoride (PMSF) protease inhibitor. Samples were subsequently boiled at 95 °C for 15 minutes and centrifuged twice at 15,000 X g for 10 min. Protein concentration and qualitative immunodetection of MatK was accomplished as described by Barthet and Hilu [12]. In brief, protein concentration was determined by Bradford assay [75] and total protein resolved by SDS-PAGE followed by transfer to nitrocellulose membrane. Transfer and loading efficiency was determined by Ponceau S staining. Bound anti-MatK antibody [12] was detected using chemiluminescent detection with HRP-conjugated anti-rabbit IgG as the secondary antibody.

Orchids used for Western blot analysis included species from two different subfamilies (Orchidoideae: *S. vernalis, S. cernua*, *S. sinensis*, *C. catenata,* and *C. erecta* and Epidendroideae: *P. tancarvilleae*) and are representative of MatK protein requiring translation from the aic (Fig. 2a and b). Our choice of species was constrained by availability of orchid tissue from commercial vendors or botanic collections. Western blot experiments for *P. tancarvilleae*, *S. vernalis* and *S. cernua* were repeated using three biological replicates whereas a single biological replicate was used for *C. erecta*, *C. catenata* and *S. sinensis*. The last three orchid taxa, *C. erecta*, *C. catenata* and *S. sinensis*, are native to Australia with limited commercial or botanic availability outside of Australia limiting tissue availability for biological replicates. *Oryza sativa* protein extract was used as a positive control for antibody binding [12].

### RNA Extraction/ RT-PCR/ MatK Splicing Activity

Leaf tissue was harvested from three biological replicates each of *S. vernalis, S. cernua* and *P. tancarvilleae*. Frozen tissue for each biological replicate was ground under liquid nitrogen and total RNA extracted using Qiagen Plant RNAeasy kit (Qiagen). Residual DNA was removed by DNA digest using Ambion’s Turbo DNAse I (Ambion). First strand synthesis was accomplished using Superscript III First Strand Synthesis Kit (Invitrogen) and an oligo dT_(20)_ primer. Removal of DNA from RNA preparations was determined by PCR on RNA subjected to the first strand synthesis protocol lacking Superscript III reverse transcriptase (no-RT control). First strand cDNA was amplified using primers NITtrnkRev (5’- GGTTGCTAACTCAACGGTAGAG-3’) and trnK 3’end primer (5’-GGGACTCGAACCCGGAA-3’) (this study). Amplified product size was used to determine MatK splicing activity. Unspliced product would result in a band of ~2833 base pairs (bp) while spliced product would result in a band of 50 to 61 bp depending on species. PCR products were resolved on 1.7 % agarose gels stained with Ethidium Bromide and visualized on a UV transluminator. PCR products indicative of matured *trnK* were gel excised and extraneous components removed using the QIAquick Gel Extraction kit (Qiagen) followed by sequencing to confirm identity.

### Availability of supporting data

The data set used for phylogenetic analysis supporting results of this article is available in Dryad, DOI number: doi: 10.5061/dryad.j4489 [76].

## Additional files

Additional file 1: Table S1. Listing of all species and associated accessions used in the phylogenetic analysis and evolution of the alternative initiation codon. (PDF 316 kb) Additional file 2: Figure S1. The nucleotide sequence alignment of the 5’ end of the *matK* open reading frame for 104 Orchidaceae and outgroup monocot taxa. Alignment was manually carried out in QuickAlign [66] with gaps being introduced at the cost of two or more substitutions. (a) The 5’ region of *matK* in all orchids with sequence extending beyond the conserved initation codon. (b) Alignment highlighting the use of the consensus (cic) vs. the alternative initiation codon (aic) in orchids and related outgroup species. Species that use the cic have a gap added to the extreme 5’ end of the *matK* sequence. The gray arrow indicates the aic while the black arrow indicates the cic. The special case of *Neottia nidus-avis* which produces a full-length *matK* ORF using the −6 in-frame initiation codon is highlighted in red. Outgroup monocots are indicated with a bracket. (PDF 264 kb) Additional file 3: Figure S2. A RAxML phylogeny of over 100 taxa in the Orchidaceae based on *matK* open reading frame nucleotide sequences computed in the CIPRES portal (http://www.phylo.org) [70] applying the default settings and conducting 1000 replicates. The tree is rooted with members of the Asparagales (monocot) families Asteliaceae (*Astelia alpina*, *Milligania stylosa*), Blandfordiaceae (*Blandfordia grandiflora*), Boryaceae (*Borya septentrionalis*), Hypoxidaceae (*Hypoxis hemerocallidea*, *Spiloxene serrate*) and Lanariaceae (*Lanaria lanata*). Taxa names are abbreviated following the limits set in the programs for character number. A summary tree of this data is provided in the main manuscript as Fig. 6. (PDF 308 kb) Additional file 4: Figure S3. Alignment of current accessions available for *Phaius tancarvilleae* in GenBank. Initiation codon required for full-length MatK translation is noted to the right of the alignment: aic = alternative initiation codon, cic = consensus initiation codon. Nucleotides are color coded: adenine = yellow; thymine = red; guanine = green; cytosine = blue. (PDF 92 kb) Additional file 5: Table S2. Source of plant material used in molecular assays. (PDF 922 kb)
