# Supplementary material for: Alternative translation initiation codons for the plastid maturase MatK: unraveling the pseudogene misconception in the Orchidaceae
Source: BMC Evol Biol. 2015 Sep 29;15:210. doi: 10.1186/s12862-015-0491-1 (PMC4587860; doi:10.1186/s12862-015-0491-1)
Supplement: Additional file 1: Table S1. — Listing of all species and associated accessions used in the phylogenetic analysis and evolution of the alternative initiation codon. (PDF 316 kb) [file 12862_2015_491_MOESM1_ESM.pdf]

Additional File 1: Table S1.

Listing of all species and associated accessions used in the phylogenetic analysis and evolution of the alternative initiation codon. Subfamily and tribe affiliation in the Orchidaceae as well as type of initiation codon required for translation of full-length *matK* ORF are provided.

Species	Subfamily	Tribe	Accession Number	Initiation Codon Type[1]
**ORCHIDACEAE**				
Apostasia nipponica	Apostasioideae		AY557215.1	cic
Apostasia nuda	Apostasioideae		AY557214.1	aic
Apostasia odorata [2]	Apostasioideae		AY557213.1	cic
Apostasia wallichii	Apostasioideae		AY557212.1	cic
Apostasia sp . G244[2]	Apostasioideae		JN181464.1	cic
Neuwiedia borneensis	Apostasioideae		AY557209.1	cic
Neuwiedia veratrifolia	Apostasioideae		AY557211.1	cic
Neuwiedia zollingeri	Apostasioideae		AY557210.1	cic
Cypripedium calceolus	Cypripedioideae	Cypripedieae	AY557208.1	aic
Paphiopedilum glaucophyllum	Cypripedioideae	Cypripedieae	AY557205.1	aic
Phragmipedium kovachii	Cypripedioideae	Phragmipedieae	AY918826.1	aic
Selenipedium aequinoctiale	Cypripedioideae	Selenipedieae	EF079360.1	aic
Bletilla striata	Epidendroideae	Arethuseae	EF079331.1	aic
Coelogyne barbata	Epidendroideae	Arethuseae	AF302720.1	aic
Entomophobia kinabaluensis	Epidendroideae	Arethuseae	AF302697.1	aic
Pleione delavayi	Epidendroideae	Arethuseae	AF503731.1	aic
Govenia sp.	Epidendroideae	Calypsoeae	EF525690.1	aic
Chytroglossa aurata	Epidendroideae	Cymbidieae	FJ564753.1	aic
Cryptocentrum calcaratum	Epidendroideae	Cymbidieae	DQ210970.1	cic
Cuitlauzina candida	Epidendroideae	Cymbidieae	EF079217.1	aic
Cymbidium atropurpureum	Epidendroideae	Cymbidieae	AF470465.1	aic
Gomesa concolor	Epidendroideae	Cymbidieae	FJ564816.1	aic
Grandiphyllum auriculum	Epidendroideae	Cymbidieae	FJ564947.1	aic

Heterotaxis violaceopunctata	Epidendroideae	Cymbidieae	DQ210678.1	cic
Hofmeisterella eumicroscopia	Epidendroideae	Cymbidieae	FJ565091.1	aic
Leochilus inconspicuus	Epidendroideae	Cymbidieae	FJ564943.1	cic
Maxillaria camaridii	Epidendroideae	Cymbidieae	DQ210724.1	cic
(=Brassia garayana)				
Miltonioides sp.	Epidendroideae	Cymbidieae	EF079210.1	aic
Oliveriana brevilabia	Epidendroideae	Cymbidieae	FJ564814.1	aic
Paphinia seegeri	Epidendroideae	Cymbidieae	EF079236.1	aic
Pescatoria lehmannii	Epidendroideae	Cymbidieae	EF079240.1	aic
Raycadenco ecuadorensis	Epidendroideae	Cymbidieae	FJ565127.1	aic
(=Fernandezia ecuadorensis)				
Rodriguezia pulchra	Epidendroideae	Cymbidieae	FJ564960.1	aic
Dendrobium findleyanum	Epidendroideae	Dendrobieae	EF079348.1	aic
Arpophyllum giganteum	Epidendroideae	Epindendreae	AF265485.1	aic
Bletia catenulata	Epidendroideae	Epindendreae	AY121718.1	cic
Cattleya acklandiae	Epidendroideae	Epindendreae	AF263810.1	aic
Dilomilis montana	Epidendroideae	Epindendreae	AF263765.1	aic
Dimerandra emarginata	Epidendroideae	Epindendreae	AF263784.1	aic
Lanium avicula	Epidendroideae	Epindendreae	AF263778.1	aic
(=Epidendrum avicula)				
Leptotes bicolor	Epidendroideae	Epindendreae	AF263789.1	aic
Masdevallia pinocchio	Epidendroideae	Epindendreae	AF265445.1	aic
Octomeria gracilis	Epidendroideae	Epindendreae	AF265484.1	aic
Pleurothallis amparoana	Epidendroideae	Epindendreae	AF265467.1	aic
Pseudolaelia vellozicola	Epidendroideae	Epindendreae	AF263776.1	aic
Restrepia aristulifera	Epidendroideae	Epindendreae	AF265481.1	aic
Restrepiopsis striata	Epidendroideae	Epindendreae	AF265480.1	aic
(=Vanda striata)				
Sophronitis cernus	Epidendroideae	Epindendreae	EF079310.1	aic
Stelis argentata	Epidendroideae	Epindendreae	AF265464.1	aic

Trisetella scobina	Epidendroideae	Epindendreae	AF265449.1	aic
Cephalanthera longibracteata	Epidendroideae	Neottieae	HM640666.1	cic
Neottia nidus-avis	Epidendroideae	Neottieae	EF079303.1	cic
Palmorchis trilobulata	Epidendroideae	Neottieae	AJ310052.1	cic
Eria javanica	Epidendroideae	Podochileae	EF079354.1	aic
Thelasis carinata	Epidendroideae	Podochileae	AY121736.1	aic
Ceratocentron fesselii	Epidendroideae	Vandeae	AB217715.1	aic
Micropera pallida	Epidendroideae	Vandeae	AB217738.1	aic
Acampe ochracea	Epidendroideae	Vandeae	AB217701.1	aic
Aerides krabiensis	Epidendroideae	Vandeae	EF655784.2	aic
Cleisomeria pilosulum	Epidendroideae	Vandeae	AB217718.1	aic
Hygrochilus parishii	Epidendroideae	Vandeae	AB217733.1	aic
Lesliea mirabilis (= *Phalaenopsis mirabilis*)	Epidendroideae	Vandeae	AB217735.1	aic
Phalaenopsis amabilis	Epidendroideae	Vandeae	AB217747.1	aic
Pomatocalpa diffusum	Epidendroideae	Vandeae	AB217752.1	aic
Sarcoglyphis comberi	Epidendroideae	Vandeae	AB217759.1	aic
Trudelia pumila (=*Vanda pumila*)	Epidendroideae	Vandeae	AB217770.1	aic
Aa hartwegii	Orchidoideae	Cranichideae	AM900803.1	aic
Chloraea gaudichaudii	Orchidoideae	Chloraeeae	AJ310004.1	aic
Codonorchis lessonii	Orchidoideae	Codonorchideae	AJ310008.1	aic
Codonorchis PJK-2001	Orchidoideae	Codonorchideae	AJ310007.2	aic
Anoectochilus koshunensis	Orchidoideae	Cranichideae	EU797512.1	aic
Aulosepalum hemichreum	Orchidoideae	Cranichideae	AM884247.1	aic
Deiregyne riodelayensis	Orchidoideae	Cranichideae	AM884237.1	aic
Eurystyles borealis	Orchidoideae	Cranichideae	AJ543925.1	aic
Galeottiella sarcoglossa	Orchidoideae	Cranichideae	AJ543945.1	aic
Goodyera viridiflora	Orchidoideae	Cranichideae	AJ310035.1	aic

Manniella gustavi	Orchidoideae	Cranichideae	AJ543944.1	aic
Pachyplectron arifolium	Orchidoideae	Cranichideae	AJ310051.1	aic
Pterostylis longifolia (=Bunochilus longifolia)	Orchidoideae	Cranichideae	AJ310062.1	aic
Sarcoglottis acaulis	Orchidoideae	Cranichideae	AJ310068.1	aic
Spiranthes praeclara	Orchidoideae	Cranichideae	AJ310073.1	aic
Corycium excisum	Orchidoideae	Diseae	EU301534.1	aic
Disa glandulosa	Orchidoideae	Diseae	AJ310021.1	aic
Disperis lindleyana	Orchidoideae	Diseae	AY370652.1	aic
Pterygodium cleistogamum	Orchidoideae	Diseae	EU687539.1	aic
Acianthus confusus	Orchidoideae	Diurideae	AJ309999.1	aic
Adenochilus nortonii	Orchidoideae	Diurideae	AJ309995.1	aic
Caladenia barbarossa	Orchidoideae	Diurideae	AJ310026.1	aic
Caleana mjor	Orchidoideae	Diurideae	AJ310001.1	aic
Chiloglottis trapeziformis	Orchidoideae	Diurideae	AJ310003.1	aic
Corybas neocaledonicus	Orchidoideae	Diurideae	AJ310011.1	aic
Cyanicula gemmata	Orchidoideae	Diurideae	AJ310017.1	aic
Diuris sulphurea	Orchidoideae	Diurideae	AJ310024.1	aic
Eriochilus cucullatus	Orchidoideae	Diurideae	AJ310028.1	aic
Genoplesium fimbriatum	Orchidoideae	Diurideae	AJ310031.1	aic
Leptoceras menziesii	Orchidoideae	Diurideae	AJ310039.1	aic
Lyperanthus serratus	Orchidoideae	Diurideae	AJ310040.1	aic
Megastylis gigas	Orchidoideae	Diurideae	AJ310042.1	cic
Microtis parviflora	Orchidoideae	Diurideae	AJ310045.1	aic
Praecoxanthus aphyllus	Orchidoideae	Diurideae	AJ310057.1	aic
Rimacola elliptica	Orchidoideae	Diurideae	AJ310066.1	aic
Thelymitra carnea	Orchidoideae	Diurideae	AJ310076.1	aic
Cleistes rosea	Vanilloideae	Pogonieae	AJ310006.1	aic
Pogonia ophioglossoidesgi	Vanilloideae	Pogonieae	AJ310055.1	aic

Epistephium sp.	Vanilloideae	Vanilleae	EF06502.1	aic
Vanilla planifolia	Vanilloideae	Vanilleae	AJ310079.1	aic
**OUTGROUP**				
Milligania stylosa	Asteliaceae	-	HM640649.1	cic
Astelia alpina	Asteliaceae	-	AY368372.1	cic
Blandfordia grandiflora	Blandfordiaceae	-	JX903609.1	cic
Borya septentrionalis	Boryaceae	-	HM640651.1	cic
Spiloxene serrata	Hypoxidaceae	-	JX903621.1	cic
Hypoxis hemerocallidea	Hypoxidaceae	-	HM640657.1	cic
Lanaria lanata	Lanariaceae	-	AY368376.1	cic

[1]Initiation codon used for translation of full-length MatK ORF is abbreviated as alternative initiation codon (aic) or consensus initiation codon (cic). The -6 initation codon used by *Neottia nidus-avis* is noted as the cic as it is in frame with the cic for translation.

[2]Additional species/accessions only used to discern *matK* evolution in the Apostasioideae and were not included in the phylogenetic analysis.
