# Supplementary figures and images for: Alternative translation initiation codons for the plastid maturase MatK: unraveling the pseudogene misconception in the Orchidaceae
Source: BMC Evol Biol. 2015 Sep 29;15:210. doi: 10.1186/s12862-015-0491-1 (PMC4587860; doi:10.1186/s12862-015-0491-1)

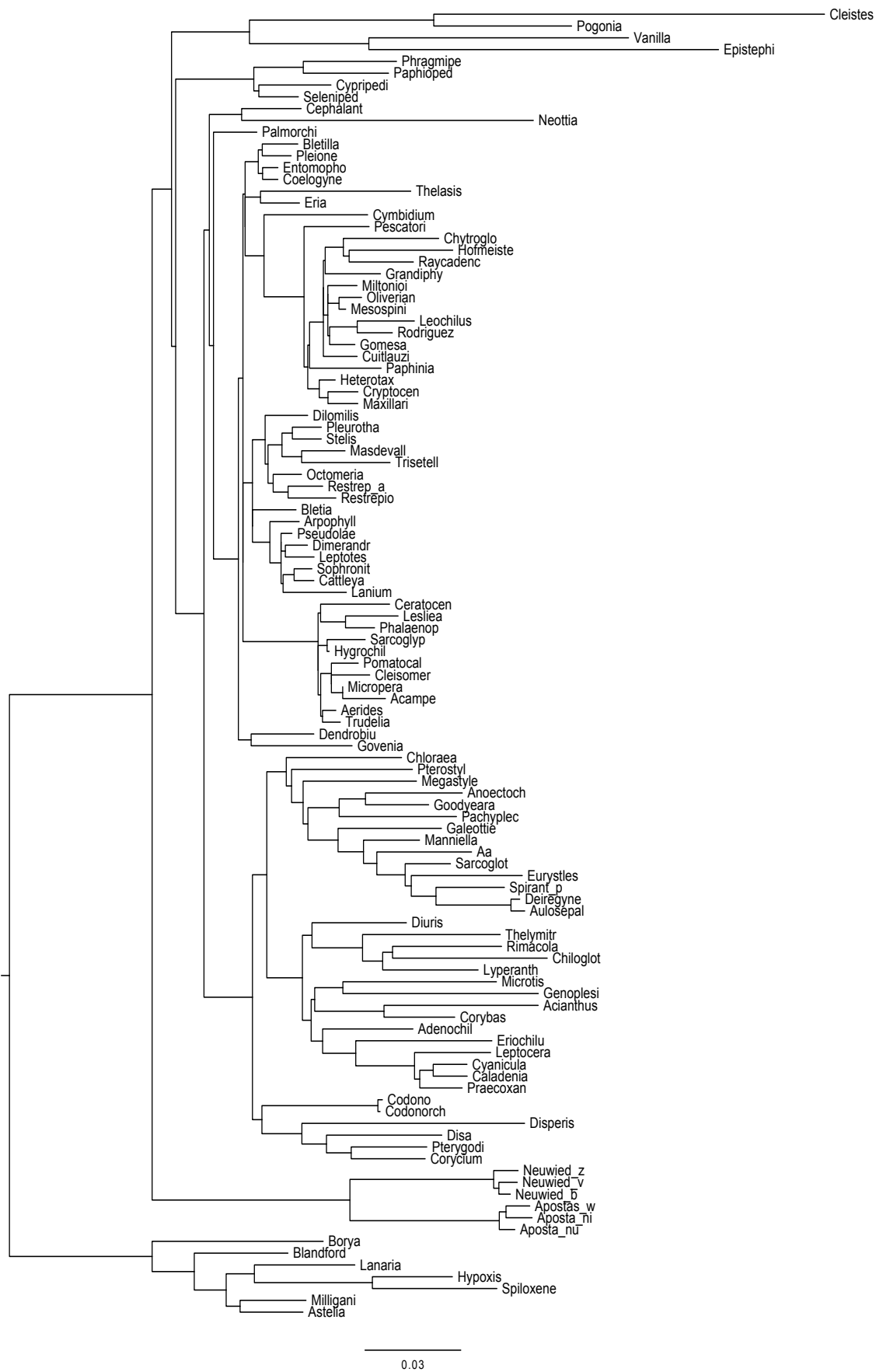

Additional file 3: Figure S2.

Supplement: Additional file 2: Figure S1. — The nucleotide sequence alignment of the 5’ end of the matK open reading frame for 104 Orchidaceae and outgroup monocot taxa. Alignment was manually carried out in QuickAlign [66] with gaps being introduced at the cost of two or more substitutions. (a) The 5’ region of matK in all orchids with sequence extending beyond the conserved initation codon. (b) Alignment highlighting the use of the consensus (cic) vs. the alternative initiation codon (aic) in orchids and related outgroup species. Species that use the cic have a gap added to the extreme 5’ end of the matK sequence. The gray arrow indicates the aic while the black arrow indicates the cic. The special case of Neottia nidus-avis which produces a full-length matK ORF using the −6 in-frame initiation codon is highlighted in red. Outgroup monocots are indicated with a bracket. (PDF 264 kb) [file 12862_2015_491_MOESM3_ESM.pdf]

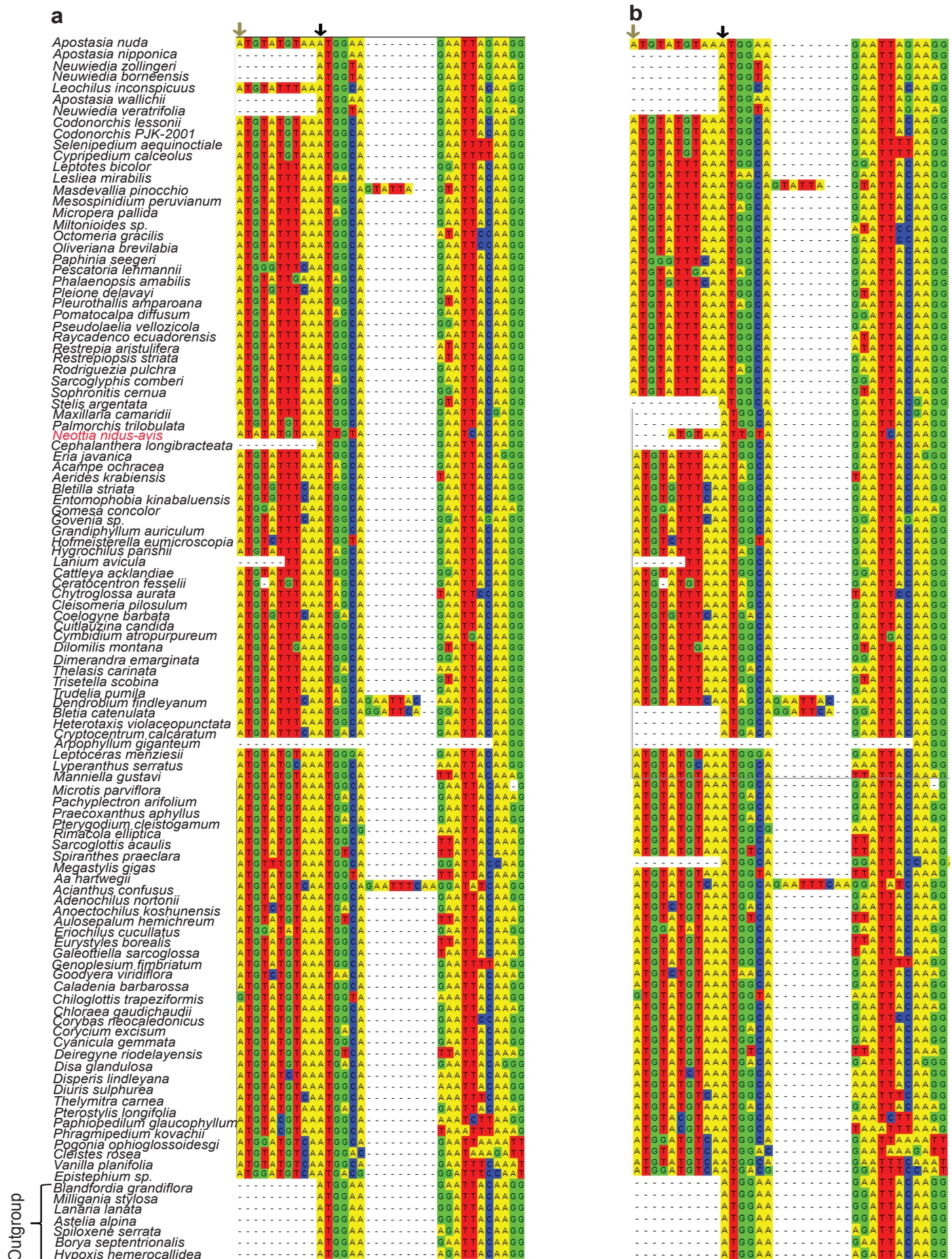

Additional file 2: Figure S1.

Supplement: Additional file 5: Table S2. — Source of plant material used in molecular assays. (PDF 922 kb) [file 12862_2015_491_MOESM2_ESM.pdf]
