# Supplementary material for: Alternative translation initiation codons for the plastid maturase MatK: unraveling the pseudogene misconception in the Orchidaceae
Source: BMC Evol Biol. 2015 Sep 29;15:210. doi: 10.1186/s12862-015-0491-1 (PMC4587860; doi:10.1186/s12862-015-0491-1)
Supplement: Additional file 3: Figure S2. — A RAxML phylogeny of over 100 taxa in the Orchidaceae based on matK open reading frame nucleotide sequences computed in the CIPRES portal (http://www.phylo.org) [70] applying the default settings and conducting 1000 replicates. The tree is rooted with members of the Asparagales (monocot) families Asteliaceae (Astelia alpina, Milligania stylosa), Blandfordiaceae (Blandfordia grandiflora), Boryaceae (Borya septentrionalis), Hypoxidaceae (Hypoxis hemerocallidea, Spiloxene serrate) and Lanariaceae (Lanaria lanata). Taxa names are abbreviated following the limits set in the programs for character number. A summary tree of this data is provided in the main manuscript as Fig. 6. (PDF 308 kb) [file 12862_2015_491_MOESM4_ESM.pdf]

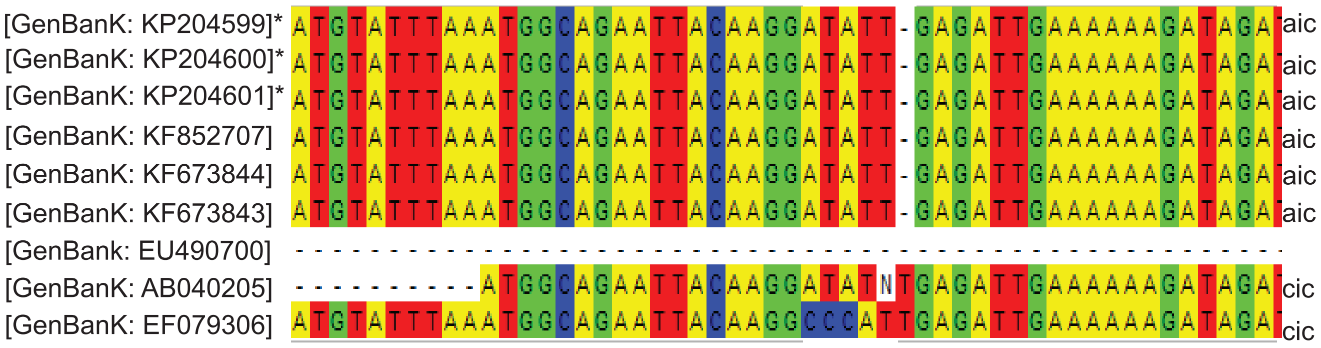

Additional file 4: Figure S3.
