# Supplementary material for: Alternative translation initiation codons for the plastid maturase MatK: unraveling the pseudogene misconception in the Orchidaceae
Source: BMC Evol Biol. 2015 Sep 29;15:210. doi: 10.1186/s12862-015-0491-1 (PMC4587860; doi:10.1186/s12862-015-0491-1)
Supplement: Additional file 4: Figure S3. — Alignment of current accessions available for Phaius tancarvilleae in GenBank. Initiation codon required for full-length MatK translation is noted to the right of the alignment: aic = alternative initiation codon, cic = consensus initiation codon. Nucleotides are color coded: adenine = yellow; thymine = red; guanine = green; cytosine = blue. (PDF 92 kb) [file 12862_2015_491_MOESM5_ESM.pdf]

Additional file 5: Table S2

Source of plant material used in molecular assays.

Orchid species	Source
Spiranthes vernalis	Field collection, Conway, SC, USA (Voucher: CCUMMB0001)
Spiranthes cernua var. oderata	Hirt's Garden, private vendor, Granger Township (Medina), Ohio
Phaius tancarvilleae	Brookgreen gardens, Murrell's Inlet, SC, USA
Spiranthes sinensis	Mount Annan, Royal Botanic Gardens, Sydney, NSW, Australia
Caladenia catenata	Mount Annan, Royal Botanic Gardens, Sydney, NSW, Australia
Cryptostylis erecta	Mount Annan, Royal Botanic Gardens, Sydney, NSW, Australia
Phaius australis	Mount Annan, Royal Botanic Gardens, Sydney, NSW, Australia
Pterostylis saxicola	Mount Annan, Royal Botanic Gardens, Sydney, NSW, Australia
